# When, where and how should we assess professionalism in undergraduate medical education? Practical tips from an international conference roundtable discussion

**DOI:** 10.12688/mep.20532.1

**Published:** 2024-11-18

**Authors:** Caitlin Patterson, Alexandra Goodwin, Kathleen Collins, Scott Oliver, Catherine Paton

**Affiliations:** 1 Medical Education, NHS Lanarkshire, Bothwell, Scotland, UK; 2 Paediatrics, Western Health and Social Care Trust, Londonderry, County Londonderry, UK; 3 Medicine, University of Glasgow College of Medical Veterinary and Life Sciences, Glasgow, Scotland, UK

**Keywords:** Professionalism, Undergraduate, Assessment, Hidden Curriculum, Professional, Pedagogy, International

## Abstract

The assessment of professionalism in healthcare disciplines is a challenging and nuanced topic in medical education. The literature, although continually emerging, remains in its infancy in regards to the role of the assessment of professionalism, appropriate timing for assessment, methods to assess professionalism and the benefits and implications of assessment of professionalism.

With emergence of healthcare professionalism in both undergraduate and postgraduate curricula and increasing awareness of professionalism’s pertinent role in developing as a healthcare practitioner, the concept of assessing this topic is being discussed regularly in international fora, but as yet there is no consensus decision in how best to proceed.

The authors have over a decade of experience researching, promoting and delivering healthcare professionalism education. They presented a roundtable discussion to an international panel of medical educators at an international conference. The attendees represented multiple healthcare disciplines. Breakout rooms and pre-determined introductory questions were used to explore the international consensus on current thinking about assessment in healthcare professionalism.

This paper presents these findings as practical tips for educators who are considering introducing or extending their assessment of undergraduate professionalism, all of which were taken from the main themes of the discussion. The aim of the paper is to support educators to think about their stance on this often divisive issue, consider their approaches and focus future research to clarify the remaining unknowns.

## Introduction

Professionalism in healthcare is defined by the authors as behaviours, attributes and actions exhibited and upheld by a healthcare professional, which result in good clinical practice and facilitate trusting relationships with patients and caregivers.

Historically most undergraduate medical students learnt about professionalism from the hidden curriculum, but in recent times it has emerged in the formal curricula of most United Kingdom (UK) medical schools ^1^. This is indicative of professionalism’s importance in the journey a clinician takes from novice to expert during undergraduate study and postgraduate practice. The literature is ever widening around the topic; in correlation the conversation around the need and ways to assess it, is ever present in educational spheres ^2– 4^.

Despite modernised thinking, emerging research and current understanding, healthcare professionalism remains primarily associated with negative connotations ^5, 6^. Anecdotally, teaching and learning around professionalism typically focuses on the avoidance of ‘unprofessional’ behaviour and discussion of regulations and sanctions that ensue in the context of professionalism lapses ^7, 8^. As follows, current assessment strategies for professionalism often take the form of global rating scales regarding student conduct, and scoring systems where points are collected if there are lapses in professionalism.

Professionalism now also features explicitly in many summative assessments for undergraduates in the UK; and is prominent in the UK Medical Licensing Assessment (UKMLA) ^9^. Improving understanding of the most appropriate ways to assess professionalism must now be considered.

## Methods

A roundtable discussion was presented, by the authors, at the 9th International Clinical Skills Conference (ICSC9), Monash University Prato Centre, in Prato, Italy on 23 ^rd^ May 2023 ^10^. The authors sought to consider how professionalism might be comprehensively assessed, the relevant pedagogy of assessment of professionalism and the purpose of professionalism assessment. The international audience offered an innovative opportunity to harbour experience and opinion from across the world.

The roundtable session was set in the wider context of ICSC9 where the theme was “Together’, with a focus on diversity and inclusion in healthcare professions education. Conference delegates could view the session abstracts via the conference website and booklet. Participants self-selected their choice of activities and opted in to participation in this discussion, which was delivered in parallel with various other sessions across the conference venue.

The roundtable participants comprised approximately 40 highly experienced, senior clinical educators drawn from a diverse range of international medical schools including Australia, New Zealand, the United States (USA), United Kingdom, Denmark, Sweden and Canada. Participants’ roles included senior doctors, nurses, allied health professionals and medical education academics.

Participants were welcomed to the roundtable and introduced to the plan for the session. Participants were split into three groups. The facilitators (CP, CP, AG, SO) rotated between each group to discuss the questions below. Facilitators recorded notes summarising ideas discussed. The following pre-defined follow-up questions were used to develop the discussion within each group:

1. What methods should we use to assess professionalism?
2. When in the year should we assess professionalism?
3. Where in a career should we assess professionalism?

The groups then reformed for feedback to the larger group. A large group discussion was facilitated by the presenters to collectively explore experience of and actions to address the challenges of and barriers to assessing professionalism in healthcare education. All participants gave written consent to share any ideas that were discussed in the roundtable.

The roundtable discussion led to fruitful conversations between educators with variable experience in conducting assessments of professionalism. The debate and discussion surrounding the complexities and barriers surrounding the assessment of professionalism as well as enabling factors and sage advice have been organised into a series of practical tips that could be incorporated into the reader’s educational practice.

### Practical tips

***Integrate professionalism into formal curriculum prior to assessment***. Before assessment can even be considered, careful consideration has to be given to the curriculum in which the assessment will reflect.

The delegates were clear that in order to be able to assess professionalism, it was paramount that it was reflected in formal medical school curricula. Consensus was reached that it would only be acceptable to assess topics that feature in the explicit programme of learning. This aligns with published literature discussing how professionalism might optimally integrate into undergraduate medical school curricula ^11, 12^.

Discussions explored the need to strike a balance between explicit professionalism teaching and integrative learning through a spiral curriculum. Explicit teaching was necessary to ensure learners understood the importance of appreciating and exploring these topics. However, to isolate professionalism as standalone lectures, or in ‘miscellaneous’ areas of the curriculum, could underemphasise its nuances and complexity, compromising authenticity and potentially generating distress for educators and learners. It was widely agreed that to assess professionalism, it would have to be integrated into all clinical themes.

Participants also discussed that professional identities and norms vary within and between medical disciplines. Assessment processes must account for this as students progress across the breadth and depth of their undergraduate course. Moreover, expectations of ‘professionalism’ could mean different things at different stages and trajectories of career and clinical experience. This must also be considered in the design of any overarching professionalism assessments.

Delegates emphasised the importance of implementing professionalism assessments in a manner sympathetic to its complexities. Some attendees reported experience of failed and/or pedagogically unsound attempts to introduce professionalism into curricula and exams. They implored colleagues to avoid taking such mis-steps due to the significant distress this had caused their students and fellow educators.

Drawing upon this discussion it is suggested that professionalism curricula should be broadly agreed and aligned with regulatory guidance, practical complexities and career stage of learners within each healthcare discipline. Professionalism teaching should be integrated across the undergraduate curriculum and learners given the opportunity to learn the facts and experience the complexity before summative assessments are considered.

***Consider professionalism as a journey of discovery that requires reflection and scaffolding by senior clinicians***. It was acknowledged that “learning professionalism” is quite different to much of the content learning in medical school; it cannot be simply memorised like the clotting cascade. Professionalism is formed through behaviours and attitudes that are acquired over time, continually fluctuate and change based on age, stage and experience and emerge through a process of discovery and reflection.

Vygotsky coined the notion that learning occurs in a zone of proximal development, scaffolded by an expert teacher ^13^. In practice this sometimes means that students learn about professionalism through their role models. Assessment should focus on key experiences, highlighted by those in trusted role modelling positions, at opportune times. Delegates felt this would support students to become actively conscious of professionalism, how it impacts their actions, interactions and stimulates their moral compass and offer a more real reflection of their professionalism.

Roundtable participants expressed that opportunities to assess professionalism could be embedded through self-led reflection. Reflective learning would need to be taught and understood to ensure success, self-actualisation and moral awareness. Discussion around the reflections can be revisited alongside the students, thus further embed the learning and understanding, founded in their own personal interpretations of a situation.

It was acknowledged, however, that pressures on clinical time may impact upon the available space for clinician role models to facilitate such teaching and assessment. Anecdotally and in the authors’ collective experience, such time pressures are a barrier to medical education in general. The authors postulate that professionalism may be more vulnerable than traditional clinical teaching in this context. It was also suggested that this may lead to inequity of opportunity for learners if clinical attachments were not truly equivalent across different peripheral sites.

***The assessment of professionalism should be longitudinal (throughout undergraduate and postgraduate careers)***. Delegates shared overwhelming consensus that snapshot examinations at single time points (i.e. in final year undergraduate exams) could not represent an individual’s overall understanding or capacity for professionalism ^1^. Binary pass/fail outcomes were firmly felt to be detrimental to the assessment of professionalism; longitudinal, summative assessment was considered paramount:

*“Professionalism is a virtue that is successively built upon. A strategy for assessment of professionalism should incorporate regular reviews of professional behaviours, from the first year of the course, to foster an understanding of professionalism early that can be regularly built upon.”*
*“Every interaction, every learning activity- promotes self-awareness [of professionalism].”*

There was broad consensus that longitudinal assessment processes enable consistently poor professionalism to be addressed in greater context and offer multiple opportunities for intervention. This was felt to offer a more authentic representation of the clinician, whilst allowing for social and professional maturation, development of professional identity formation and opportunities to rectify lapses with support and signposting.

It was also agreed that assessment should stretch beyond undergraduate careers and be lifelong. While outside the scope of this particular roundtable discussion, this is an area deserving of further exploration.

These views align with the literature pertaining to professionalism. Numerous assessment tools are described, but most rely upon snapshot judgements or assessments that occur remotely from the students’ actual clinical interactions with patients or clinicians ^14– 16^. Expert opinion concurs with the views expressed during our discussion that longitudinal assessment tools anchored in core clinical activity are much better suited to assessing professionalism ^1^.

***Dedicate time to develop clinicians to contribute to shop-floor assessments and provide structured frameworks for additional support***. Participants felt it was likely that many front-line clinicians would feel unsure how to discuss or teach professionalism to students. They suggested this could mean there were missed opportunities to scaffold development in professionalism. A lack of consensus around the threshold standards students should demonstrate during clinical placements could compound this difficulty. Some attendees also reported uncertainties or ambiguity about when or how to discuss concerns about individual students with faculty leads.

It was agreed that faculty development is required for educationalists and front-line teacher clinicians to support both student and teacher to “feel safe” in the context of assessments of professionalism. This must be buffered by transparent, fair and robust support systems from the university with clear outcomes shared. In return, students should be aware of escalation procedures, and supported in turn to escalate any concerns they had about professionalism scenarios they had witnessed during the course of their clinical placements.

***Marking schemes, if used, should be values-based***. Participants felt that a tapestry of multiple assessments, for example, a combination of summative clinical placement assessments, OSCE examinations and outcomes in written examinations, would provide the most accurate reflections of professionalism. They also felt strongly that the evaluation process should be values-based.

There was considerable discussion about how “single” adequate professionalism could be defined, if not through binary, “tick box” marking. Participants felt a global rating scale such as a marking schema or grid should be used to guide towards successful acquisition of professionalism skill being assessed. Attendees reflected on experience of using “tick box” assessment charts, and the acceptance that this system can be cheated or overcome. It was lamented that most experienced educators had encountered learners who could tick the correct boxes in an assessment context, but who demonstrated consistently poor professionalism in day-to-day activities. This is also reported in the literature ^1^.

It was felt that marking scheme should include combinations of elements that represented achievement of positive and avoidance of negative professionalism attributes. For example: awareness of behaviours, inclusion and diversity; satisfactory attendance and engagement, and meeting deadlines. Though delegates felt that such criteria should be values based, the definition of these values was not explored in detail and is an interesting area for future research. In particular, the literature reports potential dissonance between personal and (educational or clinical) institutional values, and a paucity of work relating to those of patients, carers and the general public ^17^.

Educators also considered that professionalism assessment results should be fed back and explained to students. Ideally, this would be facilitated by an experienced clinician educator through a feedback conversation, with the intention of stimulating reflection and self-actualisation.

***Consider ethical and cultural bias, patient and family perspectives and neurodiversity when formulating assessment criteria***. The audience reflected key elements that were required to successfully embed professionalism assessments. They should be free from bias. They should not discriminate against protected characteristics. They should also be inclusive for neurodivergent individuals. Assessors must have awareness of, and acknowledge, personal biases.

This presents many challenges, particularly in relation to the fluid landscape of what constitutes “acceptable professionalism” in the modern age. It was noted that inter-generational gaps between acceptable professional behaviour and modern moral foundations may not always align. Participants highlighted the potential for this to create an “assessment void” between assessors and students. Suggested solutions to these barriers included: an emphasis on longitudinal assessment, ensuring that no individual assessor has undue control, gathering evidence from different contexts, individuals and in different clinical and social environments and using defined assessment criteria that minimise the potential for bias.

The importance and value of patient, carer and public perspectives was discussed at length. Each of these groups were considered likely to hold excellent insights into the students’ behaviours and actions (notwithstanding the potential for bias discussed above). It was noted that these rarely feature in professionalism assessments at present. Participants reflected that patient and public experience could be the key to creating a robust and genuinely reflective assessment of professionalism. Delegates broadly agreed that further work was urgently required to investigate optimal ways of integrating these perspectives into assessment strategies.

***Show clarity to learners about standards expect for progression through the professionalism curriculum***. Delegates also discussed the challenges of managing situations where a student failed to pass a summative assessment of professionalism. It was unanimously agreed that if concerns were raised about a student, then they should be addressed. However, the detail around this can be challenging; for example, determining the optimal setting for any discussion, its documentation and follow-up, and any sanction or remediation that should be applied. It was recognised too, that there must be clarity about faculty expectations of students at every stage of progression. Pathways for raising and addressing professionalism issues require to be visible and accessible to learners and educators at all times.

Participants felt that summative rather than formative assessment would be more likely to capture “lapses in professionalism” or failure to meet expected standards. This was considered to be helpful in potentially providing opportunities to intervene and correct perceived difficulties with professional behaviour. Many attendees felt that the manner in which such lapses were addressed was of critical importance. Responses should be supportive, not punitive, and educators should hesitate to reprimand a student without seeking detailed context and explanation.

Where students raise persistent or high level concerns, institutions must utilise a robust pathway to address the results of these assessments. Credible faculty, with the requisite knowledge and experience in professionalism matters, should support this process.

A final practical suggestion that unified delegates concerned the juxtaposition of healthcare students with postgraduate clinical practice. It was accepted that students do not “exist in a vacuum” and that a failure to adhere to professional standards may represent a current or future risk to patients. This should be kept in mind when determining how a learner should progress following professionalism concerns. Ultimately, current and future duty of care to patients must feature in this decision, and there may be instances where further progression in the course is not justified.

## Conclusion

This roundtable discussion highlighted the complexity of professionalism assessments in the context of healthcare professional education. The available literature is limited and the described practical experience illuminates a need for further work to enhance the implementation of professionalism assessments in a manner that benefits learners, teachers and, ultimately, patients.

Professionalism is an emerging field of medical education. This highly experienced, international audience of medical educators, considered that professionalism assessment was an essential component of students’ developmental journeys into mature, self-aware postgraduate professionals. Key messages arising in this meeting are outlined below in Table 1:

**Table 1.** Key Messages: Summary of recommendations from expert opinion.

	Factors to consider when planning the assessment of professionalism
1.	Professionalism requires to be formalised and visible within the curriculum before assessment is considered
2.	Expectations, standards and assessment pathways for professionalism must be fully disclosed and readily accessible to learners and educators
3.	Educators need support to develop skills in conducting assessments of professionalism
4.	Longitudinal assessment techniques are required to allow for evolving learner maturity, experience and boundaries based on current training level to be respected
5.	Escalation of professionalism issues requires a clear transparent framework
6.	Professionalism assessment is an essential component of medical education

It is clear that a substantial body of further work is required in this area of medical education. We suggest that priority is given to achieving a consensus on standards of professionalism; the optimisation of assessment methodologies; and exploring how patients, carers and the general public can contribute to this essential process.

## Ethics and consent

This study aligns with the principles of the Declaration of Helsinki. Ethical approval was not required for this study as the format is a report of a roundtable discussion using an established format within the context of an international medical education conference. At the beginning of the roundtable, all participants were made aware that facilitators would take notes summarising key points of the discussion and that this would be used to write a publication. All participants agreed and signed written consent forms. This methodology is in line with the ethical guidelines of NHS Lanarkshire.

This research was conducted in line with the principles of the Declaration of Helsinki. All participants signed consent forms to consent to the content of the roundtable discussion to be used to produce a publication. Collected data is anonymised.

## Data Availability

The written interview guide as well as field notes taken from the Roundtable discussion are available online ^18, 19^. This data is available under terms of the Creative Commons Attribution 4.0 International licence (CC-BY 4.0) ( https://creativecommons.org/licenses/by/4.0). Figshare: Interview guide - when, why, where to assess professionalism? (ICSC Prato 9, May 2023). https://doi.org/10.6084/m9.figshare.26096242.v1 ^18^. This project contains the following extended data: Interview Guide Prato Roundtable.docx Data are available under the terms of the Creative Commons Attribution 4.0 International license (CC-BY 4.0). Figshare: Field notes - roundtable discussion, ISCS9, Prato: When, where and how could professionalism be assessed? https://doi.org/10.6084/m9.figshare.27316518.v2 ^19^. This project contains the following extended data: Field notes Data are available under the terms of the Creative Commons Attribution 4.0 International license (CC-BY 4.0). Nil.
