# Supplementary material for: When, where and how should we assess professionalism in undergraduate medical education? Practical tips from an international conference roundtable discussion
Source: MedEdPublish (2016). 2024 Nov 18;14:280. [Version 1] doi: 10.12688/mep.20532.1 (PMC11803192; doi:10.12688/mep.20532.1)
Supplement: Peer review history

# Reviewer report: Reviewer response for version 1

**DOI:** 10.21956/mep.21975.r40144

**Published:** 2024-12-20

**Authors:** Patricia Neville

**Affiliations:** 1 University of Bristol, Bristol, UK

Thank you for this Practical Tips submission.  Your roundtable discussion summary provides a useful overview of the challenges faced when trying to assess healthcare professionalism.  

 Professionalism is a second order concept1 influencing and underpinning all aspects of health care delivery and education. The diffuse nature of professionalism is complicated further by its multi-dimensional qualities (knowledge, behavioural and attitudinal domains), that it is context dependent, shaped by the organizational/hierarchical spaces, practices and people health professionals interact with and observe daily, and developmental with some people not progressing in same upward trajectory as others. 

 While I disagree with the pronouncement that scholar work into the assessment of profession ‘remains in its infancy’ (a PubMed search using the crude search term ‘assessing professionalism’ yielded 147,000 plus results, dating back to the 1930s), a key question still evades the field, and this piece, namely what methods should we use to assess professionalism? Unsurprisingly, many of the systematic reviews of assessment tools in professionalism suggest that multiple tools, rather than a single assessment point, are needed to assess student professionalism.2-4  The more pressing question for educators then is how best to integrate and embed these assessment tools into the curriculum? While curriculum mapping5 the teaching and assessing of professionalism will bring visibility to the task(i.e. formal curriculum) we still need to reconcile the fact that the assessment of professionalism is a resource hungry activity, requiring time and space in the curriculum. More important is the recognition among students and faculty alike, that being professional isn’t merely compliance with a regulatory body or ticking off curricular learning outcomes; it is the acknowledgement of the everyday/micro power and privilege afforded to members of the healthcare profession and the obligation on them to employ anti-discriminatory practices (hidden curriculum).6,7.

If evidence from practice is presented, are all the underlying source data available to ensure full reproducibility?

Not applicable

Is the topic of the practical tips discussed accurately in the context of the current literature

Partly

Are all factual statements correct and adequately supported by citations?

Yes

Are the conclusions drawn balanced and justified on the basis of the presented arguments?

No

Are arguments sufficiently supported by evidence from the published literature and/or the authors’ practice?

Yes

Reviewer Expertise:

Sociology of health, sociology of professions, dental education.

I confirm that I have read this submission and believe that I have an appropriate level of expertise to confirm that it is of an acceptable scientific standard, however I have significant reservations, as outlined above.

## References

[1] : Perceptions of professionalism in dentistry – a qualitative study. *British Dental Journal* .2013;215(9) : 10.1038/sj.bdj.2013.1048 10.1038/sj.bdj.2013.1048 24201649

[2] : A blueprint to assess professionalism: results of a systematic review. *Acad Med* .2009;84(5) : 10.1097/ACM.0b013e31819fbaa2 551-8 10.1097/ACM.0b013e31819fbaa2 19704185

[3] : Assessing medical professionalism: A systematic review of instruments and their measurement properties. *PLoS One* .2017;12(5) : 10.1371/journal.pone.0177321 e0177321 10.1371/journal.pone.0177321 28498838 PMC5428933

[4] : Assessing Professionalism in Medicine - A Scoping Review of Assessment Tools from 1990 to 2018. *J Med Educ Curric Dev* .2020;7: 10.1177/2382120520955159 2382120520955159 10.1177/2382120520955159 33150208 PMC7580192

[5] : AMEE Guide No. 21: Curriculum mapping: a tool for transparent and authentic teaching and learning. *Med Teach* .2001;23(2) : 10.1080/01421590120036547 123-137 10.1080/01421590120036547 11371288

[6] : Re-opening Pandora's box: Who owns professionalism and is it time for a 21st century definition?. *Med Educ* .2023;57(1) : 10.1111/medu.14862 66-75 10.1111/medu.14862 35761477 PMC10083973

[7] : The Problem and Power of Professionalism: A Critical Analysis of Medical Students' and Residents' Perspectives and Experiences of Professionalism. *Acad Med* .2023;98(11S) : 10.1097/ACM.0000000000005367 S32-S41 10.1097/ACM.0000000000005367 37983394

# Reviewer report: Reviewer response for version 1

**DOI:** 10.21956/mep.21975.r40289

**Published:** 2024-12-13

**Authors:** Deena M. Hamza

**Affiliations:** 1 University of Alberta, Edmonton, Alberta, Canada

I commend the authors for leveraging a round table discussion that underpins the values of collective intelligence to explore professionalism from a variety of perspectives.

 The round table discussions highlight the complexity and importance of assessing professionalism in undergraduate and postgraduate training, including in unsupervised practice. The authors also describe that in order to assess professionalism, the field requires a rigorous examination of how professionalism is taught. The captured discussion illustrates the need to create an environment that provides formal and informal lessons on this topic.

 The authors also pose critical questions, such as the methods to assess professionalism, the timing of the assessment, and where in one's career should professionalism be assessed. These are all questions that, in my experience, we have yet to answer in a meaningful and cogent manner.

 Most prominent is the awareness that tick-boxes as marking schemes to assess professionalism can be cheated or overcome, and this is couched in existing evidence in the literature.

 Further, the authors embed the aspect of professionalism in broader discussions about ethical and cultural bias, patient and family perspectives, and neurodiversity when developing assessments. This holistic view is refreshing.

 Notably, the practical elements suggested by the authors through this round table discussion are not always supported by literature foundations. This is not an issue with the rigour with which the authors have examined the topic and subsequently written this paper. Rather, this reveals the needed work and advancement of scholarship to uncover and mobilize learnings about professionalism that stem from practical experience.

 One area to bolster this much-needed article is a more fulsome description of how the practical tips were generated by the authors. In the paper, there is a discussion about the summarizing of discussions and leveraging the field notes of facilitators, but how did the content, as displayed in this paper, come to be? Independent review and content analysis? Consensus on the practical tips? More clarity will provide the reader with more information to critically appraise the efforts of the authors and provide transferrable methods that others can adapt to their contexts.

If evidence from practice is presented, are all the underlying source data available to ensure full reproducibility?

Partly

Is the topic of the practical tips discussed accurately in the context of the current literature

Yes

Are all factual statements correct and adequately supported by citations?

Yes

Are the conclusions drawn balanced and justified on the basis of the presented arguments?

Yes

Are arguments sufficiently supported by evidence from the published literature and/or the authors’ practice?

Yes

Reviewer Expertise:

CBME, innovation design and implementation, evaluation methodology and methods

I confirm that I have read this submission and believe that I have an appropriate level of expertise to confirm that it is of an acceptable scientific standard, however I have significant reservations, as outlined above.

# Reviewer report: Reviewer response for version 1

**DOI:** 10.21956/mep.21975.r40142

**Published:** 2024-12-10

**Authors:** Ola Lindberg

**Affiliations:** 1 Umeå University, Umeå, Sweden

The article addresses the problems involved when assessing professionalism in medical education. Teaching and assessment often focus on avoiding "unprofessional" behaviors, relying on binary or punitive systems that fail to capture professionalism's complexities. Discussions at the 9th International Clinical Skills Conference emphasized embedding professionalism in curricula, using longitudinal assessments, and integrating reflective learning supported by senior clinicians. Addressing biases and incorporating patient perspectives were also highlighted as crucial steps. These approaches aim to foster a comprehensive understanding of professionalism, promoting resilience and authentic professional identity formation throughout a clinician's career. The discussion concluded that further work is needed to optimize assessment methodologies and ensure inclusive, equitable, and meaningful standards.

 The discussions addressed several problems hitherto explored in the literature. It gives a comprehensive overview of the issues and dilemmas involved in the assessment of professionalism. The discussions are well described and summarized, and provides an excellent overview for any scholar or practitioner interested in the issues surrounding assessment of professionalism. There are more sociological and philosophical questions that could be addressed, particularly concerning bias in assessment (who do we see as professional and why?) and the idea that professionalism is “one thing” rather than several. Viewing professionalism as contextual and cultural rather than a standardized construct might offer more understanding of the complexity involved.

If evidence from practice is presented, are all the underlying source data available to ensure full reproducibility?

Yes

Is the topic of the practical tips discussed accurately in the context of the current literature

Yes

Are all factual statements correct and adequately supported by citations?

Yes

Are the conclusions drawn balanced and justified on the basis of the presented arguments?

Yes

Are arguments sufficiently supported by evidence from the published literature and/or the authors’ practice?

Yes

Reviewer Expertise:

Professional education, workplace learning, reflection

I confirm that I have read this submission and believe that I have an appropriate level of expertise to confirm that it is of an acceptable scientific standard.
